# Action Recognition of Lower Limbs Based on Surface Electromyography Weighted Feature Method

**DOI:** 10.3390/s21186147

**Published:** 2021-09-13

**Authors:** Jiashuai Wang, Dianguo Cao, Jinqiang Wang, Chengyu Liu

**Affiliations:** 1 School of Engineering, Qufu Normal University, Rizhao 276826, China; jiashuaiw23@163.com (J.W.); 18764348818@163.com (J.W.); 2 School of Instrument Science and Engineering, Southeast University, Nanjing 210096, China; chengyu@seu.edu.cn

**Keywords:** action recognition, surface electromyography, weighted feature method, championship and sorting

## Abstract

To improve the recognition rate of lower limb actions based on surface electromyography (sEMG), an effective weighted feature method is proposed, and an improved genetic algorithm support vector machine (IGA-SVM) is designed in this paper. First, for the problem of high feature redundancy and low discrimination in the surface electromyography feature extraction process, the weighted feature method is proposed based on the correlation between muscles and actions. Second, to solve the problem of the genetic algorithm selection operator easily falling into a local optimum solution, the improved genetic algorithm-support vector machine is designed by championship with sorting method. Finally, the proposed method is used to recognize six types of lower limb actions designed, and the average recognition rate reaches 94.75%. Experimental results indicate that the proposed method has definite potentiality in lower limb action recognition.

## 1. Introduction

Muscles are an important part of the human body and provide power for locomotion [1]. Surface electromyography (sEMG) is derived from the surface of human skeletal muscle, is closely related to neuromuscular activity and contains much information related to limbs [2]. sEMG signals are acquired by placing sEMG sensors on the muscle surface and recording electrical signals [3]. sEMG has been widely used in the field of action recognition and has achieved compelling results [4,5,6,7,8]. Since sEMG involves nonstationary bioelectric signals, wearers will experience muscle fatigue, unstable sEMG signals and other interference during the signal acquisition process. Therefore, as the training time increases, the SNR will decrease and the noise will increase [9]. This requires the extraction of more representative signal features to represent the activity of muscle [10]. Many researchers use time-domain and frequency-domain analysis methods to obtain feature vectors [11,12,13]. However, pure time-domain and frequency-domain features contain less useful information to characterize the activity of muscle. Recent studies have shown that features in the time-frequency domain can effectively characterize the activity information of sEMG signals [14]. After the researchers extract the features, they usually perform dimensionality reduction processing on the features [15,16] to reduce the dimensionality of features while retaining some effective features as much as possible; this can reduce the redundancy of the feature vector, but will still discard some effective features. Therefore, the discrimination of actions and the recognition rate will be reduced. In response to the above problems, the weighted feature method based on the correlation between muscles and actions was proposed. The extracted original features of different channels are weighted differently. Therefore, the weighted features can be obtained. In the same action and different channels, the redundancy of features can be reduced. In different actions and the same channel, the discrimination of features can be increased.

**Figure 1:**
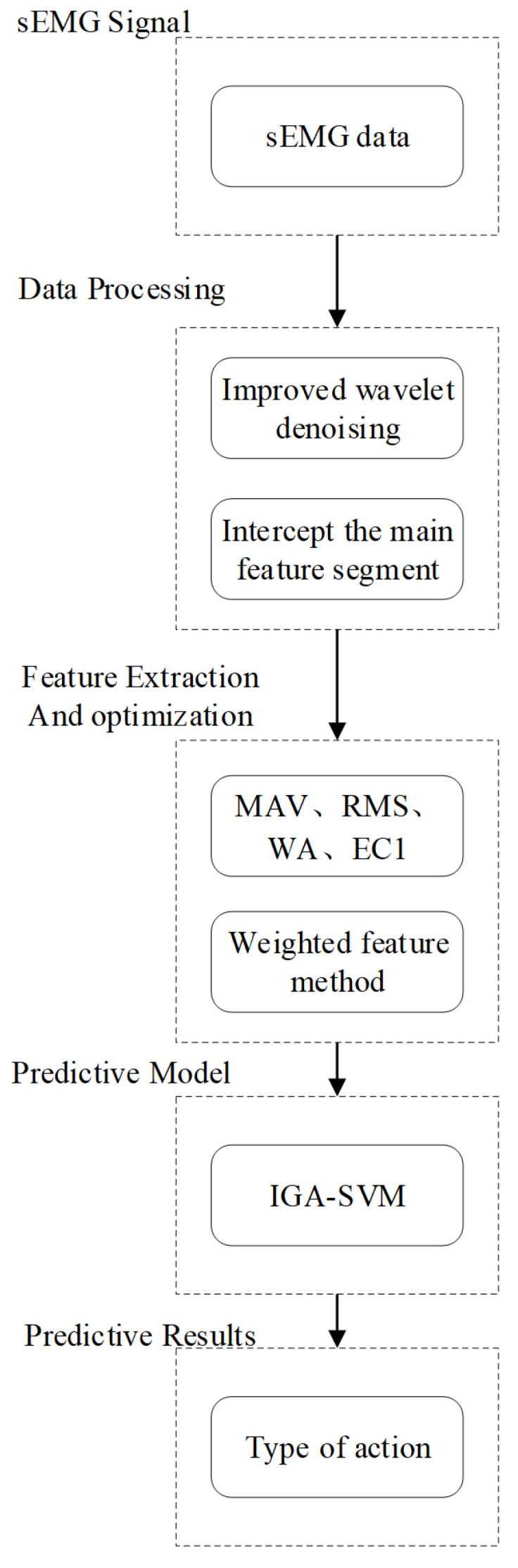
Action recognition framework.

At present, classification algorithms that are commonly used for the construction of sEMG signal classification models include linear discriminant analysis (LDA), the nonparameter estimation classifier K-nearest neighbors (KNN), SVM based on discriminant function analysis, convolutional neural networks (CNNs), etc. [17,18,19]. The LDA classifier finds the most suitable linear combination of data variables. Its calculation is easier and suitable for identifying the difference between linear samples, but it is not ideal for nonlinear classification [20]. KNN is easier to implement and has efficient computing power, but the classification recognition rate is greatly reduced when new samples of imbalanced data sets are input [21]. A CNN can automatically extract signal features and is suitable for high-dimensional data processing, but it easily falls into a local optimum. Moreover, the encapsulation of the feature extraction process increases the difficulty of improving the network [22]. SVM can solve the problems of high dimensionality and nonlinearity, and the processing process is transparent, and one of the links can be optimized and improved [23]. In view of the advantages and disadvantages of different classifiers, this paper selects SVM as the classifier and optimizes it by improving the genetic algorithm to improve the action recognition rate. The framework of the overall action recognition in this paper is shown in Figure 1, where the MAV is mean absolute value, RMS is root mean square, WA is Willision amplitude, EC1 is wavelet packet coefficient energy value, IGA-SVM is improved genetic algorithm–support vector machine classifier.

## 2. Methods

### 2.1. The Framing Energy Method Is Used to Extract the Main Feature Signals

The time for an ordinary person to complete a lower limb action is approximately 3 s. Therefore, each signal acquisition time should be as long as possible during the experiment to include all the effective signals of actions, but this will produce the disadvantages of a large amount of data and a useless resting signal. These disadvantages will increase the difficulty of signal processing. We extract the main feature segment from a complete signal to solve the problem. The main feature segment contains most of the effective information of actions and filters out of resting signals, so the timeliness of action recognition is improved [24].

During the experiment, the signal is divided into three segments: the initial segment, the main feature segment, and the ending segment. The initial segment is defined as the data segment from the beginning of the action to 0.5 s, the main feature segment is from the end point of the initial segment to the end of the following 2 s, and the ending segment follows the main feature segment. The ending segment is ignored and not processed. The initial point is detected by using the signal frame energy method [25,26]. We use 64 sampling points of the signal as the sliding window frame and 32 sampling points as the increment and calculate the adaptive threshold th through the resting signal. After that, we calculate the energy value of each frame of the signal and record it as En. Comparing En with th, when En is greater than th three times in a row, this frame is judged as the starting point of the initial segment. Suppose the signal is X(n)=x(1),x(2),...,x(N), where *N* is the sum of the data length, and the calculation method of the signal framing energy method is as follows:

(1) Select the appropriate frame length and frame shift to divide the normalized signal into frames:

(1) (M−1)×I+L=N

where *M* is the total number of frames of the signal, *I* is the incremental frame step, *L* is the sampling point of each sliding window, namely, the frame length of each frame, and *N* is the total length of the signal. The signal after obtaining the split frame is $X^{\prime }\left(n\right)=x^{\prime }\left(1\right),x^{\prime }\left(2\right),...,x^{\prime }\left(N\right)$, where $x^{\prime }\left(1\right)=x(1),...,x(L),x^{\prime }\left(2\right)=x(1+I),...,x(L+I),...$.

(2) Calculate the total energy of each frame signal:

(2) $$En\left(i\right)=\sum_{i=1}^{n+L}amp_{in}^{2}\;$$

where En(i) is the total energy of the signal per frame and $amp_{in}$ is the amplitude of the n−th sampling point in frame *i*, with n<L.

(3) Calculate the adaptive threshold th based on the signal energy when standing steadily:

(3) $$th=\frac{\sum_{i=1}^{M}En\left(i\right)}{M}\;$$

If En is greater than th in a certain frame and is greater than th in the next three frames, then this frame is the starting frame of the signal action segment.

(4) Obtain the main feature segment by extracting 2 s of data 0.5 s after the start point of the action:

(4) $$\begin{array}{c} \left\{\begin{array}{c} SN=\left(FS-1\right)\times F_{s}\\ MSN=SN+0.5\times F_{s}\\ MEN=MSN+2\times F_{s} \end{array}\right. \end{array}$$

Among them, SN is the detected sampling point at the starting point, FS is the number of frames at the starting point, $F_{s}$ is the sampling frequency, MSN is the sampling point at 0.5 s after the starting point, and MEN is the sampling point at 2.5 s after the starting point. The signal from MSN to MEN is the signal of the main feature segment.

### 2.2. Feature Extraction from the Main Feature Segment Signal

sEMG signal analysis methods mainly include time domain analysis, frequency domain analysis, parameter modeling, time-frequency analysis, nonlinear dynamic analysis and so on. Through the analysis of the differences between the physiological responses of sEMG signals [27,28], this paper selects the features in Table 1 to recognize the lower limb actions. The overall trends of the selected features under different actions are similar, but the local responses are different. Therefore, we consider extracting, optimizing and fusing the features in Table 1 as the input of the lower limb action classifier.

The calculation methods of the four features in Table 1 are as follows:

(5) $$sEMG_{MAV}=\frac{1}{M}\sum_{j=1}^{M}(\frac{1}{n-1}\sum_{i=1}^{n}|x_{i}\left|\right)\;$$

(6) $$sEMG_{RMS}=\frac{1}{M}\sum_{j=1}^{M}\sqrt{\frac{1}{n-1}\sum_{i=1}^{n}x_{i}^{2}}\;$$

(7) $$sEMG_{WA}=\frac{1}{M}\sum_{j=1}^{M}(\sum_{i=1}^{n-1}f(|x_{i}-x_{i+1}|))\;$$

(8) $$sEMG_{EC1}=\log_{10}(\frac{1}{M}\sum_{j=1}^{M}|S_{j}{|}^{2})\;$$

From (5)–(8), $x_{i}$ is the amplitude of the *i*-th sampling point of the sEMG signal, *M* is the total number of windows after signal framing, *n* is the length of the window, f(x)=1 when x≥th and f(x)=0 otherwise, and $S_{j}$ is the wavelet packet coefficient of the *j*-th window.

### 2.3. Weighted Feature Method

sEMG signals are complex and nonstationary [31], so the difference in the extracted features is small. From the perspective of feature differences, first, we calculate the signal energy value $sumabsX_{i}^{\prime }$ of each muscle under different lower limb actions and record and establish the energy table. The energy value represents the contribution degree of each muscle to different actions. Second, according to the contribution degree, we calculate the correlation degree between muscles and actions and establish the correlation degree table. Finally, according to the correlation coefficient in the correlation table, the features of each channel are given different weights to complete the purpose of feature optimization. The correlation between muscles and actions indicates that each muscle plays a different role in different actions. As mentioned in Section 1, the feature dimensionality reduction method may cause the loss of some effective information. However, using feature fusion directly can cause feature redundancy. Therefore, the weighted feature method based on the correlation between muscles and actions is proposed. The extracted original features of different channels are weighted differently, and the weighted features can be obtained. In the same action and different channels, the redundancy of features is reduced. In different actions and the same channel, the discrimination of features is increased. The specific calculation method is as follows:

(1) Remove the noise and baseline drift of the signal *X* to obtain $X^{\prime }$.

(2) Take the absolute value of signal $X^{\prime }$ to obtain $absX^{\prime }$.

(3) Calculate the energy value of each channel, that is, the sum of amplitudes, and obtain $sumabsX_{i}^{\prime }$, where *i* is 1,2,...,n, and *n* is the total number of channels. The number of channels selected in this experiment is 8, so n=8.

(4) Add up the energies of all channels to obtain sumabsX, where the weight of each channel can be calculated as follows:

(9) $$C_{i}=n\times \frac{sumabsX_{i}^{\prime }}{sumabsX},i=1,2,...,n\;$$

(5) Therefore, $Feature=\sum_{i=1}^{n}feature_{i}\times C_{i}$, where Feature is the fusion feature vector, and $feature_{i}$ is the feature vector of the corresponding channel.

### 2.4. Improved Genetic Algorithm-Support Vector Machine Classifier

To achieve classification, SVM establishes a classification hyperplane as a decision surface to maximize the isolation edge between different classes [32]. This is the core idea of SVM. Therefore, SVM uses the Lagrange multiplier method to solve the classification process and introduces a penalty factor *c*, which is responsible for controlling the intensity of punishment for incorrect demarcation points. By controlling the distance between the wrong demarcation point and its correct position, we can keep it within a reasonable range. When the value of *c* is high, the occurrence of incorrect demarcation points will be greatly reduced, but overfitting will occur. When the value of *c* is very low, this will lead to a large number of incorrect demarcation points, which will cause the training model to be unreasonable. The performance of the kernel function selected by SVM also determines the accuracy of classification, the radial basis kernel function $k\left(x_{i},x_{j}\right)=e^{-g||x_{i}-x_{j}{\left|\right|}^{2}}$ exhibits better performance [33], and the value of the kernel parameter *g* directly affects the prediction accuracy of the model. This paper combines a genetic algorithm to optimize the parameters *c* and *g*, constructs the population fitness based on the action recognition rate, and uses the ranking method on the basis of the championship method. For the sorting based on the fitness, the population is divided into four grades of good, well, medium, and bad, and the offspring are selected according to a certain proportion to carry out cross mutation and adaptively evolve the population to optimize the population and fitness. The specific implementation process is as follows:

(1) Fusion feature vector:

$E_{i}=\left[e_{i1},e_{i2},...,e_{ia}\right],i=1,2,...,n$ is used to represent the feature sample vector of the sEMG signal, *a* is the dimension of the feature vector, and *n* is the number of samples. The fusion feature vector is then:

(10) $$X={\left[E_{1},E_{2},...,E_{m}\right]}^{T}\;$$

where *m* is the number of features, *X* is divided into training set $X_{p}$ and test set $X_{T}$, $X_{p}$ is used to train the classifier, and $X_{T}$ is used to detect the classification effect of the classifier. Suppose there are *m* groups of samples in the training set $X_{p}$, and the corresponding categories are $Y_{p}$, i.e., $X_{p}={\left[x_{1},x_{2},...,x_{m}\right]}^{T}$ and $Y_{p}={\left[y_{1},y_{2},...,y_{m}\right]}^{T}$, where $y_{1}$ means raise right leg, $y_{2}$ means lower right leg, $y_{3}$ means raise left leg, $y_{4}$ means lower left leg, $y_{5}$ means sitting to standing, and $y_{6}$ means standing to sitting.

(2) Construct an improved genetic algorithm-support vector machine: GA-SVM uses the ability of the genetic algorithm to find the optimal solution to optimize the penalty factor *c* and the kernel parameter *g*, which will improve the classifier’s performance to a certain extent. However, the genetic algorithm will fall into a local optimal solution, resulting in limited improvement in the classification recognition rate. In this paper, on the basis of the characteristic of selecting the optimal solution via championship, the genetic algorithm is combined with the sorting method to improve GA-SVM, namely, IGA-SVM (improved genetic algorithm-support vector machine).

The championship execution process is to select a certain number of individuals from the parent population each time, then select the best one to enter the offspring population, and to repeat this operation until the new population reaches the size of the original population. Therefore, the main goal of the tournament selection operator is to find the optimal individual in the population. This has certain advantages, but it also presents the disadvantages of destroying the diversity of the population and reducing the ability to search for the population, thus causing the system to fall into a local optimal solution. Through integration with the sorting method, we establish a hierarchical elimination system, in which the individuals in the population are sorted according to their fitness, and the population is divided into four levels: bad, medium, well, and good. When the next generation is selected, the four levels of populations are selected according to a certain proportion. This not only ensures that the proportion of outstanding individuals in the population is large, but also maintains the diversity of the population and obtains the global optimal solution. The improvement steps are as follows:

(1) The initial population number is determined, the fusion feature vector $X_{p}$ is used as the training data of the genetic algorithm, the fitness of the individuals in the population is calculated, and the matrix that records the parameters and fitness of the population is denoted as oldpop.

(2) The individuals in the population are sorted from small to large according to their fitness values and recorded as matrix sortpop.

(3) The sorted individuals are divided into four grades: bad, medium, well, and good, expressed as $C_{bad},C_{mid},C_{well},C_{good}$ with $C=C_{bad}+C_{mid}+C_{well}+C_{good}$, where *C* is the total population number.

(4) According to the principle that the good is chosen more and the bad chosen less, the diversity of the population should be considered as much as possible, and it should be ensured that all outstanding individuals exist. The four levels of bad, medium, well, and good are selected according to the probabilities of P,P+σ,P+2σ,P+3σ(0<P<1,0<σ<1).

(5) The individuals selected are recombined to obtain a new population newpop. At this time, the individuals contained in the population are recorded as $C_{new}=\left[C_{bad}\times P\right]+\left[C_{mid}\times \left(P+\sigma \right)\right]+\left[C_{well}\times \left(P+2\sigma \right)\right]+\left[C_{good}\times \left(P+3\sigma \right)\right]$.

(6) Step (5) will discard a part of the population, resulting in an incomplete population matrix, so it is necessary to insert individuals into newpop. The insertion principle adopts the principle of survival of the fittest. If $C-C_{new}\leq C_{good}$, then $C-C_{new}$ individuals are randomly selected from $C_{good}$ to join the new population newpop; if $C-C_{new}\geq C_{good}$, then all the individuals in $C_{good}$ are input into the new population, and $C-C_{new}-C_{good}$ random individuals in $C_{well}$ are selected to be input into newpop so that the population number of newpop is *C*, and cross-mutation is performed in newpop to obtain better offspring.

(7) The 5-fold cross-validation method is used to calculate the fitness of the population, and the fitness is the IGA population fitness accuracy rate. If the fitness accuracy rate does not reach the target value, steps (2)–(6) are repeated until the fitness reaches the target value, and the value of the penalty factor *c* and the kernel parameter *g* are recorded at this time to obtain bestc,bestg.

(8)bestc,bestg are applied to the SVM to obtain the IGA-SVM training network, the best classification model Classifymodel is trained through the training set, and the test set $X_{T}$ is input into Classifymodel to obtain the best classification accuracy.

## 3. Results and Analysis

### 3.1. Experiment Procedure

**Figure 2:**
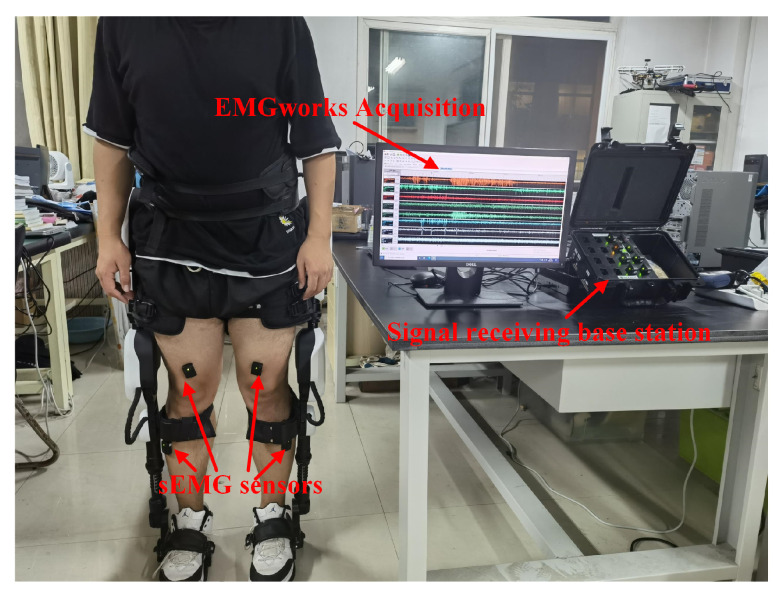
Hardware link.

**Figure 3:**
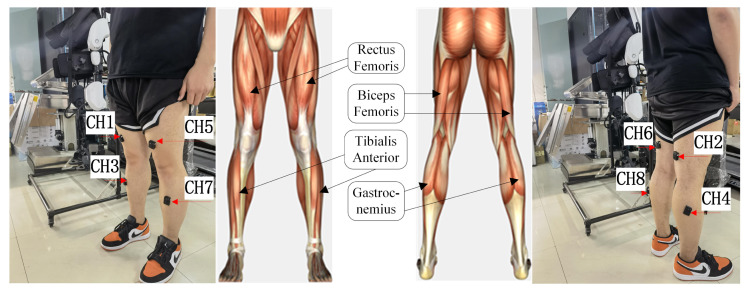
Graph of muscle positions.

At present, the known open source database does not contain the data of the six lower limb actions studied in this paper. Therefore, this paper designs an sEMG acquisition experiment. The DELSYS TrignoTM wireless sEMG signal acquisition instrument is used to acquire sEMG signals. The sampling frequency of this instrument can reach 2000 Hz. As shown in Figure 2, the hardware linked is as follows: the sEMG signals are acquired and transmitted by the sEMG sensors worn on the subjects. The sEMG signals are received by the signal receiving base station with wireless. The base station transmits the sEMG signals to PC with USB, and the PC is equipped with EMGworks Acquisition. According to the literature [34], the main sEMG features are distributed over 10–500 Hz. According to the body’s physiological structure and the distribution of leg muscles [35], we selected CH1-right leg rectus femoris, CH2-right biceps femoris, CH3-right tibial anterior muscle, CH4-right gastrocnemius, CH5-left leg rectus femoris, CH6-left biceps femoris, CH7-left tibialis anterior muscle, and CH8-left gastrocnemius as the signal acquisition sources, as shown in Figure 3. Because sEMG is susceptible to interference, volunteers needed to wipe the abovementioned muscles with alcohol before data collection and remove dander on the skin surface to reduce interference. In addition, volunteers should not exercise vigorously within 24 h before signal collection.

**Figure 4:**
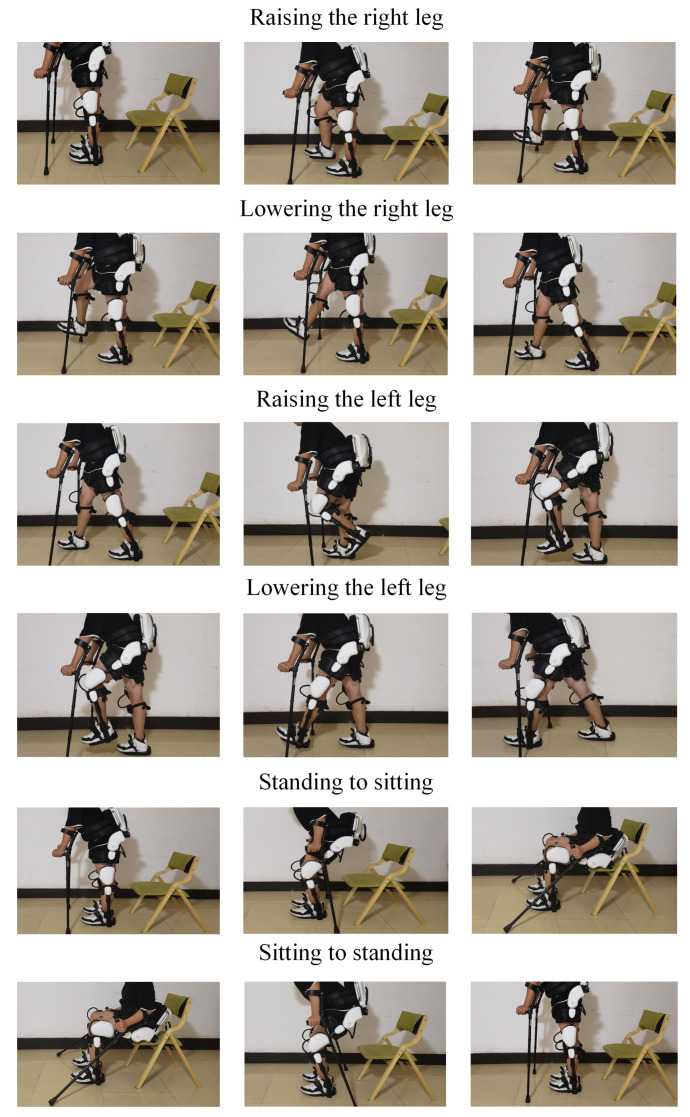
The diagram of 6 actions.

This paper studies six lower limb actions: raising the right leg, lowering the right leg, raising the left leg, lowering the left leg, sitting to standing, and standing to sitting, as shown in Figure 4. The subjects were three healthy (24–26)-year-old males (75 ± 5 kg, 175 ± 5 cm) and one healthy 25-year-old female (50 kg, 165 cm). None of them suffered from any neuromuscular system or joint diseases. All subjects volunteered to participate in this experiment and were informed of the experimental content before the experiment. To standardize the actions in the collection process, it was stipulated that when the signal was collected, subjects wearing the Fourier X2 and 8 sEMG sensors perform the above six types of lower limb actions. In the experiment process, each action is performed 500 times and each action lasted 10 s. Considering the sampling frequency of sEMG sensors is 2000 Hz, therefore, each time 8 channels acquire 8 × 20,000 data due to per channel acquires 20,000 data. These data are stored as a csv file, which is called a set of sEMG signals. By the end of experiment, 12,000 sets of sEMG signals can be obtained. Considering the fatigue of subjects during experiment, each time the subjects acquire signals within 10 min to ensure the quality of signals.

### 3.2. Analysis of Suraface Electromyography Physiological Characteristics in Action Recognition

**Figure 5:**
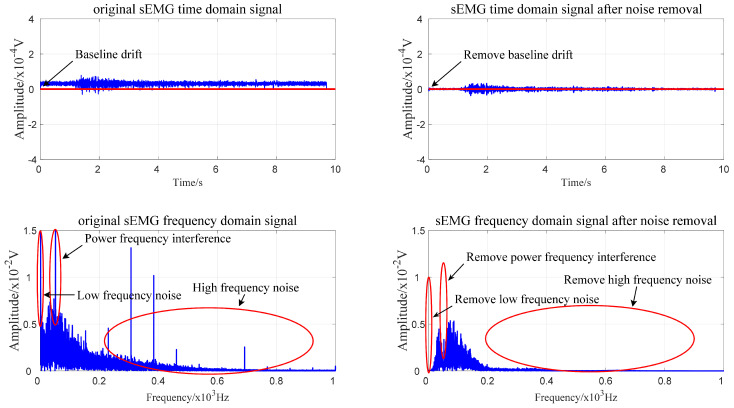
Comparison results of the time domain and frequency domain before and after wavelet denoising.

The sEMG signal is a nonstationary, nonlinear, weak electrical signal that is very susceptible to interference from environmental noise and power frequency noise. Before feature extraction and subsequent processing, it is necessary to denoise the sEMG signal and use the improved wavelet denoising method [36] to filter out the signal noise, power frequency interference and baseline drift and obtain a smooth sEMG signal with less noise. The literature [36] has already introduced improved wavelet denoising in detail, so this paper will not go into details. Here, we consider subject No. 1’s raised right leg as an example to demonstrate the processing process. The comparison results before and after denoising are shown in Figure 5.

**Figure 6:**
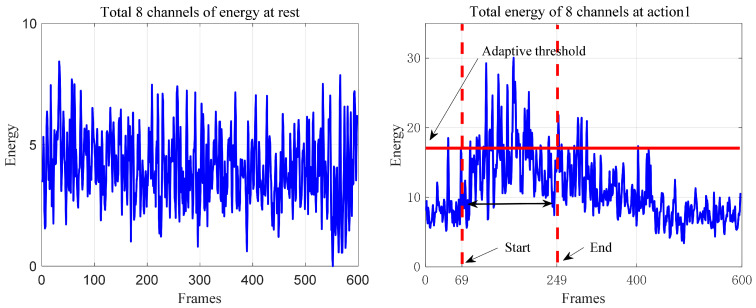
Acquisition of the data from the signal action starting point to the end point of the main feature segment.

**Figure 7:**
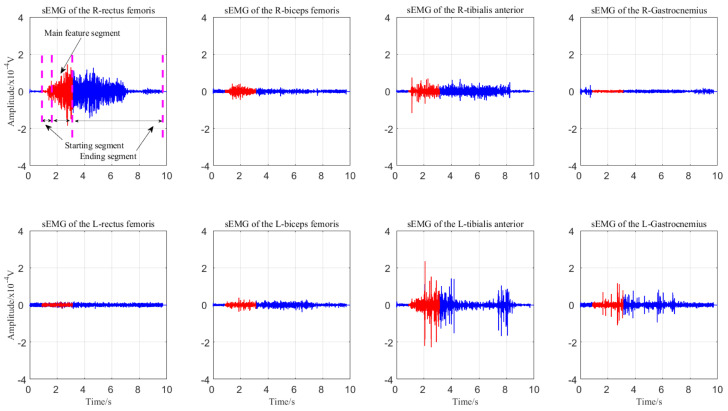
Obtaining the main feature segment of the signal.

Due to the long signal length, this paper applies the signal framing energy method to obtain the main feature segment signal with a shorter data length. First, the energy threshold of the signal is calculated, and the sum of the energy in the resting state and the sum of the energy exerted in raising the right leg are obtained, as shown in Figure 6. Second, according to the adaptive threshold, by judging that the values in three consecutive frames are greater than the adaptive threshold, this point is selected as the starting point of the action, and the data 2.5 s after the starting point are extracted. In addition, the data 0.5 s after the starting point are selected as the main feature signal segment, as shown in Figure 7.

### 3.3. Analysis of the Weighted Feature Process

The time domain features and wavelet packet coefficient energy features of the main feature signals are extracted and normalized to the interval of (−1,1), and then the correlation coefficients between muscles and actions are calculated. First, extraction of the signal of the main feature segment, calculation of the energy value of each muscle during the six kinds of actions, and establishment of an energy table were performed. As an example, for subject No. 1, the energy value is calculated with steps (1)–(3) in Section 2.3, as shown in Table 2, where 1–4 represent the signal acquisition channels of the right rectus femoris, biceps femoris, tibialis anterior muscle, and gastrocnemius muscle, and 5–8 represent the signal acquisition channels of the left leg rectus femoris, biceps femoris, tibial anterior muscle, and gastrocnemius muscle. Second, according to Table 2, steps (4)–(5) in Section 2.3 are used to calculate the correlation coefficient between muscles and actions and to establish the table of correlations, as shown in Table 3. The greater the correlation coefficient is, the greater the correlation among the muscles and the actions.

**Figure 8:**
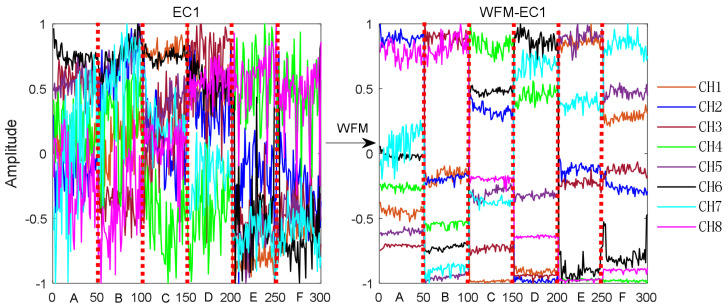
Comparison of energy features of wavelet packet coefficients of 8 channels before and after WFM.

For simplicity, we only select 50 sets of data for each of the six actions to show the energy features of the normalized wavelet packet coefficients, as shown in Figure 8. EC1 represents the energy features of wavelet packet coefficients, and WFM-EC1 represents the energy features of wavelet packet coefficients after optimization. The x-axis coordinates indicate A—raise right leg, B—lower right leg, C—raise left leg, D—lower left leg, E—sitting to standing, and F—standing to sitting, and it can be seen from the figure that EC1 has high feature redundancy and low discrimination under 6 actions with 8 channels. After WFM, WFM-EC1 has low redundancy and increased discrimination under different actions, which helps improve the recognition rate.

### 3.4. Analysis of the Optimization Process of the Improved Genetic Algorithm

**Figure 9:**
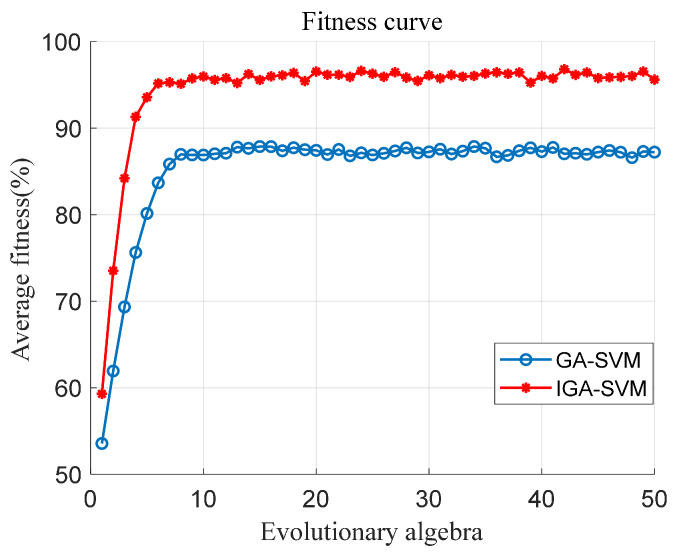
Convergence curve comparison chart of the population average fitness.

The genetic algorithm is optimized through the combination of the championship method and sorting method. To expand the diversity and universality of the population, the population size is selected as C=2000, the crossover probability is 0.7, the mutation probability is 0.1, and the population selection probability parameter is set to P=0.4, σ=0.2. A total of 12,000 sets of data samples (2000 sets for each action) are selected from the collected data for preprocessing and feature extraction. Each action takes 1/2 of the sets of data as the training set, and the 5-fold cross-validation method is used to calculate the fitness accuracy of the population to obtain the best population *newpop*. The convergence process of the population is shown in Figure 9. The population evolution generation stops at 50 iterations, and the average fitness of IGA is significantly higher than that of GA.

## 4. Discussion

Each subject produced 250 groups for each action, a total of 6000 groups (1500 groups for each subject) were used as the training set data, and the remaining data were used as the test set to verify the action recognition rates of different methods. First, we use the traditional lower-limb action recognition method to identify and classify the six kinds of lower-limb actions. Inputting the fusion feature vector without WFM into GA-SVM, the classification results are shown in Table 4.

Second, the traditional GA-SVM classifier is used to classify the fusion feature vectors after WFM, and the results are shown in Table 5. By comparison with Table 4, the recognition rates of the six lower limb actions increase by 6.7%, 9.9%, 7.7%, 3.4%, 3.3%, and 5.9%, and the average recognition rate increases by 6.15%. The experimental results show that the WFM can improve the recognition rate of the six types of lower limb actions effectively in the data set of this paper.

Third, we input the fusion feature vector without WFM into IGA-SVM for classification, and the classification results obtained are shown in Table 6. By comparison with Table 4, the recognition rates of the six lower limb actions increase by 1.8%, 2.3%, 1%, 1.3%, 0.8% and 4.6%. The results indicate that the IGA-SVM can improve the recognition rate of lower limb actions slightly in the data set of this paper.

Finally, the IGA-SVM classifier is used to classify the WFM fusion feature vector, and the results are shown in Table 7. The average recognition rates of the six actions of raising the right leg, lowering the right leg, raising the left leg, lowering the left leg, sitting to standing, and standing to sitting are 94.3%, 95.9%, 96.2%, 96.6%, 94.5% and 91%, respectively, and the average recognition rate of all actions is 94.75%. By comparison with Table 4, the method proposed in this paper increases the recognition rates of the six lower limb actions by 9.3%, 13.3%, 10.3%, 7.8%, 6.1%, and 9.8%, and the average recognition rate is increased by 9.44%. The result suggests that the WFM and IGA-SVM have a good performance in lower limb recognition, and the improvement of recognition rate is relatively obvious in the data set of this paper. Meanwhile, Table 8 shows the classification results of the test set, the number of six lower limb actions recognized are 980, 984, 981, 981, 1075 and 999. It can be seen that the difference between the results is small. From Table 8, we can also see that the actions of recognizing errors are in two similar actions, such as action5 and action6. The reason for this result may be that both actions involve the same muscles and the sEMG signals acquired are correlated. To further improve the recognition rate, the correlation can be eliminated by improving the algorithm or fusing other signals.

**Figure 10:**
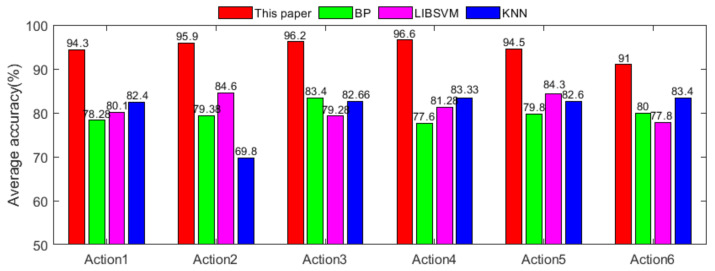
Comparison of recognition rates of different classification methods.

To further prove the effectiveness of the method proposed in this paper, we select the most commonly used classification methods in action recognition, the BP neural network, LIBSVM, and KNN [37,38], to classify the experimental data. According to the experiment, the average classification recognition rates of the three classification methods for the six lower extremity actions are 79.74%, 81.22% and 80.69%, respectively. As shown in Figure 10, the recognition rates of the method proposed in this paper for the six lower limb actions are higher than those of the above three classification methods, so the method of this paper is more suitable for the classification of lower limb actions.

## 5. Conclusions

From the perspective of action recognition in lower limb rehabilitation training, taking the six actions of raising the right leg, lowering the right leg, raising the left leg, lowering the left leg, sitting to standing, and standing to sitting as the recognition targets of lower limb actions. We propose a lower limb action recognition method based on weighted features. First, after optimizing the features of the extracted main feature segment signal, the redundancy of the feature vector is reduced, and a feature vector with greater discrimination is obtained. Second, we design an improved genetic algorithm-support vector machine classifier, obtain the global optimal solution, and establish a multi-sEMG feature fusion action recognition model. After the WFM and IGA-SVM mentioned in this paper, the recognition rates of the six lower limb actions are 94.3%, 95.9%, 96.2%, 96.6%, 94.5%, and 91%, and the average recognition rate of all actions is 94.75%. Compared with the unimproved classification method and the commonly used classification method in Figure 10, WFM and IGA-SVM obtain better recognition accuracy and performance. The experimental results show that the weighted feature method of features and improved genetic algorithm-support vector machine classification can improve the recognition rate of lower limb actions in the data set of this paper.

The method designed in this paper is mainly aimed at improving the recognition rate of lower limb movements. The next step is to take into account non-ideal factors such as muscle fatigue and electrode offset to improve the recognition rate of lower limb actions under non-ideal conditions, and to apply the method to wearable lower limb exoskeleton robots to contribute to the field of rehabilitation treatment. In addition, similar actions, such as sitting and standing, may have different EEG signals. We will explore whether the recognition rate is improved by fusing EEG signals.

## Figures and Tables

**Table 1:** Action recognition-related physiological features of sEMG signals.

Features	Feature Description
$sEMG_{MAV}$	Mean absolute value
$sEMG_{RMS}$	Root mean square
$sEMG_{WA}$	Willison amplitude
$sEMG_{EC1}$	Wavelet packet coefficient energy [29,30]

**Table 2:** Energy table.

Energy Value	Channel of the Acquisition Signal							
	1	2	3	4	5	6	7	8
action1	4.4075	7.3995	3.3853	3.0290	2.5047	10.0337	6.0779	6.1198
action2	5.6125	4.2509	16.7769	2.2035	1.9899	5.0036	2.7665	6.7932
action3	2.7713	6.8533	4.1197	8.5512	3.7452	16.5496	5.3684	3.8048
action4	3.7745	2.8812	2.6002	8.2039	4.2894	25.1282	12.0314	2.5562
action5	31.3369	13.8150	23.4856	2.1398	18.2272	14.9905	24.0917	2.6756
action6	23.3024	12.5947	25.4307	2.0793	15.1958	19.6991	29.0533	3.3588

**Table 3:** Correlation between muscles and actions.

Correlation Coefficient	Channel of the Acquisition Signal							
	1	2	3	4	5	6	7	8
action1	0.8208	1.3780	0.6304	0.5641	0.4664	1.8686	1.1319	1.1397
action2	0.9890	0.7491	2.9565	0.3883	0.3507	0.8817	0.4875	1.1971
action3	0.4283	1.0592	0.6367	1.3216	0.5788	2.5577	0.8297	0.5880
action4	0.4913	0.3750	0.3384	1.0678	0.5583	3.2706	1.5659	0.3327
action5	1.9172	0.8452	1.4368	0.1309	1.1151	0.9171	1.4739	0.1637
action6	1.4262	0.7708	1.5564	0.1273	0.9300	1.2056	1.7781	0.2056

**Table 4:** Recognition rate of the fusion feature vector without the WFM using GA-SVM classification.

Actions	Subject ID				Average Accuracy
	1	2	3	4	
action1	86.8	85.2	75.6	92.4	85
action2	84	80	79.6	86.8	82.6
action3	94	81.6	85.2	82.8	85.9
action4	94.8	93.6	87.6	79.2	88.8
action5	92.4	95.6	81.2	84.4	88.4
action6	66.4	76.8	91.6	90	81.2
Average accuracy	86.4	85.46	83.46	85.93	85.31

**Table 5:** Recognition rate of the fusion feature vector with the WFM using GA-SVM classification.

Actions	Subject ID				Average Accuracy
	1	2	3	4	
action1	95.2	93.2	92	86.4	91.7
action2	94.8	94	90.8	90.4	92.5
action3	96.8	95.2	93.2	89.2	93.6
action4	94.4	92	95.2	87.2	92.2
action5	95.2	89.2	90	92.4	91.7
action6	84.4	86.4	84.4	93.2	87.1
Average accuracy	93.46	91.66	90.93	89.8	91.46

**Table 6:** Recognition rate of the fusion feature vector without the WFM using IGA-SVM classification.

Actions	Subject ID				Average Accuracy
	1	2	3	4	
action1	90.4	86.4	76.4	94	86.8
action2	88.4	85.2	78	88	84.9
action3	94.4	81.6	87.2	84.4	86.9
action4	95.2	94	89.2	82	90.1
action5	91.2	95.2	84	86.4	89.2
action6	70.8	86.8	93.2	92.4	85.8
Average accuracy	88.4	88.2	84.66	87.86	87.28

**Table 7:** Recognition rate of the fusion feature vector with the WFM using IGA-SVM classification.

Actions	Subject ID				Average Accuracy
	1	2	3	4	
action1	97.2	95.2	95.6	89.2	94.3
action2	97.6	96.4	94.8	94.8	95.9
action3	98	97.6	96	93.2	96.2
action4	98.4	96.8	98.8	92.4	96.6
action5	97.6	92	93.2	95.2	94.5
action6	87.6	91.6	88.8	96	91
Average accuracy	96.06	94.93	94.53	93.46	94.75

**Table 8:** The results statistics of the fusion feature vector with the WFM using IGA-SVM classification.

Actual Actions	Predicted Actions						Total Number of Actions
	action1	action2	action3	action4	action5	action6	
action1	943	2	6	3	24	22	1000
action2	18	959	3	1	16	3	1000
action3	0	9	962	0	18	11	1000
action4	5	0	0	966	7	22	1000
action5	14	0	10	0	945	31	1000
action6	0	14	0	11	65	910	1000
Total number of actions	980	984	981	981	1075	999	6000
